# Catheter Ablation of Persistent AF—Where are We Now?

**DOI:** 10.31083/j.rcm2412339

**Published:** 2023-11-30

**Authors:** Louisa O’Neill, Benjamin De Becker, Maarten A.J. De Smet, Clara Francois, Jean-Benoit Le Polain De Waroux, Rene Tavernier, Mattias Duytschaever, Sebastien Knecht

**Affiliations:** ^1^Department of Cardiology, AZ Sint-Jan Hospital, 8000 Bruges, Belgium; ^2^Department of Cardiology, Blackrock Clinic, A94 E4X7 Dublin, Ireland

**Keywords:** persistent atrial fibrillation, catheter ablation, novel technology, clinical outcomes

## Abstract

Persistent atrial fibrillation (AF) is a diverse condition that includes various subtypes and underlying causes of arrhythmia. Progress made in catheter ablation technology in recent years has significantly enhanced the durability of ablation. Despite these advances however, the effectiveness of ablation in treating persistent AF is still relatively modest. Studies exploring the mechanisms behind persistent AF have identified substrate-driven focal and re-entrant sources within the atrial body as crucial in sustaining AF among individuals with persistent AF. Furthermore, the widespread adoption of atrial late gadolinium enhancement cardiac magnetic resonance (CMR) imaging and the ongoing refinement of invasive voltage mapping techniques have allowed for detailed assessment of fibrotic remodelling prior to or at the time of procedure. Translation into clinical practice, however, has yielded overall disappointing results. The clinical application of AF mapping in ablation procedures has not shown any substantial advantages beyond the use of pulmonary vein isolation (PVI) alone and adjunct ablation of fibrotic areas has yielded conflicting results in recent randomized trials. The emergence of pulsed field ablation represents a welcome development in the field and several studies have demonstrated an enhanced safety profile and increased procedural efficiency with this non-thermal energy modality. Pulsed field ablation also holds promise for safe and efficient substrate ablation beyond the pulmonary veins, but further trials are needed to assess its impact on longer term success rates. Continued advancements in our comprehension of AF mechanisms, alongside ongoing developments in catheter technology aimed at safe formation of transmural lesions, are essential for achieving better clinical outcomes for patients with persistent AF.

## 1. Introduction

Atrial fibrillation (AF) is the most prevalent cardiac arrhythmia globally and is expected to experience a twofold increase in prevalence from 2010 to 2060. By that time, it is estimated that approximately 17.9 million adults will be suffering from AF [1]. Traditional classification of AF is based on episode duration; “paroxysmal AF” is used to describe episodes that either resolve spontaneously or with intervention within a period of 7 days while “persistent AF” refers to episodes that last longer than 7 days [2]. Catheter ablation has been widely adopted as an integral part of rhythm management for AF and high 1-year procedural success rates are achievable with pulmonary vein isolation (PVI) in the paroxysmal AF cohort using current workflows [3]. Replicating these results in the persistent AF population has been significantly more challenging with the majority of studies suggesting single procedure success rates of only 50–60% at one year [4, 5, 6, 7, 8] and a considerable number of patients require more than one procedure to sustain sinus rhythm. These patients often exhibit advanced structural and electrical remodeling, allowing for ongoing initiation and perpetuation of atrial fibrillation outside the pulmonary veins. As such, adjunct ablation strategies have been proposed to target this arrhythmia substrate including linear ablation, fibrosis-based ablation and ablation of complex fractionated electrograms and rotors [9, 10, 11, 12]. Nevertheless, evidence for a consistent benefit with these strategies is lacking [5, 6, 8] and PVI remains the cornerstone of ablation as reflected in current guidelines [2]. While results from randomized controlled trials have been largely disappointing, ongoing developments in ablation technique and technology hold promise and a paradigm shift towards earlier referral and treatment may translate into better results in this population. Within this review, we outline the progression in strategy for catheter ablation of persistent AF over the last twenty years. Additionally, we emphasize recent advancements in catheter and ablation technology that show promise in enhancing outcomes for this particularly challenging patient group.

## 2. Evolution of Catheter Ablation and Mapping Techniques 

### 2.1 Pulmonary Vein Isolation

PVI represents the cornerstone of AF ablation worldwide with a class I (level of evidence A) recommendation from current guidelines [2]. As per a survey conducted in 2015, PVI was adopted as the primary procedural strategy for persistent AF patients in over 60% of the 30 European centers participating [13]. It is worth noting that the current focus on PVI signifies a shift towards a simpler approach following the extensive exploration of various additional ablation strategies over the past two decades. Described initially by Haïssaguerre *et al*. [14] early studies of PVI only in persistent AF patients yielded largely underwhelming results with higher recurrence rates compared to those with paroxysmal AF [15, 16, 17]. The inferior outcomes observed in the persistent AF cohort may be attributed to a more advanced atrial substrate, distinct from trigger-driven AF commonly observed in the paroxysmal pattern of disease, that responds favorably to PVI. In order to target this arrhythmia substrate, additional ablation techniques were proposed as additional strategies beyond PVI.

### 2.2 Linear Ablation

An adjunct strategy of linear mitral isthmus and left atrial roof ablation to achieve bidirectional conduction block, in addition to PVI, was first reported in 2004 [9, 18]. The rationale behind this approach, was derived from knowledge gained from the surgical maze procedure and concept of atrial compartmentalization, by which lines of electrical block would be theorized to reduce the overall electrical conducting area of the atria and thus limit its capacity to sustain AF [19]. This strategy gained significant traction in the face of several studies demonstrating higher success rates with linear ablation compared to PVI alone, an effect that was most notable in persistent AF patients [20, 21, 22]. These studies emphasized the difficulty in achieving acute mitral block, however, with often low final rates of bidirectional block in this region. The rate of achieving roofline block was notably greater in comparison. Although adjunct linear ablation may reduce the incidence of recurrent atrial tachycardia (AT), in the presence of incomplete block the risk of AT appears to increase [23]. In general, while some studies indicate a potential incremental benefit of linear ablation in patients with persistent AF, evidence is conflicting, and disappointing long-term outcomes have been observed in other studies [5, 21]. The randomized STAR AF II trial (Approaches to Catheter Ablation for Persistent Atrial Fibrillation) conducted in 2015, involving 589 patients across multiple centers, stands as the most rigorous assessment to date of an adjunctive ablation strategy in patients with persistent AF [5]. At 18 months no benefit was observed beyond PVI in those undergoing mitral and roof lines with only 46% free from recurrent AF. It is worth noting that block across both mitral and roof lines was achieved in only 75% of patients in this study, however and overall, the efficacy and long-term success of linear ablation as an adjunctive strategy for persistent AF patients remain uncertain.

### 2.3 Posterior Left Atrial Wall or ‘Box’ Isolation 

In 2007, Kumagai *et al*. [24] introduced the concept of box isolation as a linear, end-point driven ablation technique targeting the posterior left atrial wall. The objective of box isolation was to isolate focal and re-entrant drivers responsible for persistent AF, which were frequently identified in this region [25]. A mechanistic study utilizing phase mapping in patients with persistent AF provided support for the rationale behind the box isolation technique and revealed a decrease in left atrial critical mass and AF drivers after isolation of the posterior wall was achieved [26]. Similar to the mitral isthmus, achieving isolation of the posterior box can be challenging, however, and while this approach was not evaluated in STAR AF II, several randomized studies have shown no additional benefit to this strategy beyond PVI only [27, 28, 29]. Recently the multicentre, randomized CAPLA trial (Effect of Catheter Ablation Using Pulmonary Vein Isolation With vs Without Posterior Left Atrial Wall Isolation on Atrial Arrhythmia Recurrence in Patients With Persistent Atrial Fibrillation) addressed this question in a larger population of 338 persistent AF patients [6]. Once again, no difference in arrhythmia free survival at one year was seen in patients undergoing PVI alone vs PVI plus posterior box isolation with modest success rates seen in both groups (53.6 vs 52.4%, *p* = 0.98). Lack of benefit may relate to the iatrogenic creation of pro-arrhythmic substrate due to incompletely ablated tissue and/or failure to achieve complete isolation. Concerns regarding ablation at the posterior wall relate to its close proximity to the oesophagus, with oesophageal injury and the rare but often fatal atrio-oesophageal reported in the setting of extensive posterior wall radiofrequency (RF) ablation [5, 30, 31]. Indeed, intra-oesophageal temperature rise resulting in operator reluctance to persist with ablation is a common reason for failure to achieve complete isolation. This in turn is associated with posterior wall reconnections and treatment failure [32]. As such it carries only a IIB level of evidence C recommendation [33].

### 2.4 Complex Fractionated Atrial Electrograms (CFAE) Ablation

CFAE are characterized by the presence of complex fractionated potentials, continuous electrical activity, or short mean cycle lengths [34]. These electrograms are often observed in areas of slow conduction, conduction block, as well as at anchor points for re-entry circuits, wavefront collision, and ganglionated plexi [10, 35, 36]. In 2004, success rates from ablation of CFAE sites (mapped during AF), without PVI, were reported by Nademanee *et al*. [10] in both paroxysmal and persistent AF patients. While promising results were seen initially, a subsequent study failed to replicate these findings, with only 33% of persistent patients maintaining sinus rhythm at a follow-up of over 1 year [37]. Moreover, when CFAE ablation was performed in addition to PVI, several randomized trials reported mixed results in the persistent AF population [38, 39, 40]. The inconsistency in defining CFAEs and endpoints among different studies poses a significant challenge when comparing their results. Furthermore, mechanistic studies have suggested that a substantial portion of CFAEs might not actively contribute to the perpetuation of atrial fibrillation AF [41], leading to potential concerns regarding excessive ablation of noncritical regions. Consequently, this approach can prolong procedure times without a clearly defined objective. Adjunct CFAE ablation was evaluated in STAR AF II, again without a significant demonstrable benefit beyond PVI alone and only 49% undergoing this strategy free of recurrent arrhythmia on 18-month follow up [5].

In 2005, Haissaguerre *et al*. [42] described a ‘stepwise’ approach for persistent AF patients, combining PVI, CFAE ablation, and linear mitral and roof ablation. The procedural endpoint of this approach was AF termination. Several studies reported high rates of sinus rhythm maintenance after multiple procedures and over longer-term follow-up of up to 5 years [43, 44]. Although the STAR AF II trial did not evaluate this specific approach, proponents of the method argued that extensive substrate ablation over multiple procedures could achieve high long-term success rates in restoring sinus rhythm.

### 2.5 Mapping of AF 

The development of various advanced mapping techniques for characterization of mechanisms of persistent AF coincided with the assessment of the aforementioned ablation strategies. Phase mapping is a mathematical technique utilized to characterize spatial and temporal patterns of electrical activity in cardiac tissue. Its application to ablation of AF involves identifying and mapping periodic rotations, known as “rotors”, which play a crucial role in sustaining AF [45]. An invasive endocardial mapping approach, “focal impulse and rotor modulation” (FIRM), employs a specialized catheter with 64 electrodes arranged in a basket configuration (FIRMap by Topera, Palo Alto, CA, USA; Constellation by Boston Scientific, Marlborough, MA, USA). This technique enables the creation of endocardial maps illustrating AF propagation, facilitating real-time identification and ablation of stable foci exhibiting certain spatial and temporal characteristics [46]. The FIRM mapping approach was evaluated in the CONFIRM (Conventional Ablation for Atrial Fibrillation With or Without Focal Impulse and Rotor Modulation) trial in predominantly persistent AF patients undergoing conventional ablation vs conventional plus FIRM guided ablation [11]. The elimination of stable rotors or focal impulses through ablation led to the termination or deceleration of AF in 86% of participants. Notably, the single-procedure success rates were significantly higher in individuals who underwent adjunct FIRM-guided ablation compared to those who did not (82.4% vs 44.9%, *p* < 0.001) [47, 48]. Nevertheless, replicating these findings proved challenging [47, 48], and the randomized REAFFIRM trial (Prospective randomized comparisonof rotor ablation vs conventional ablation for treatment of persistent atrial fibrillation), which involved 375 patients with persistent AF, failed to demonstrate any outcome benefit after one year [49].

Electrocardiographic imaging (ECGi) mapping is a non-invasive form of phase mapping employing a multi-electrode body surface vest in conjunction with thoracic imaging. By analyzing unipolar electrograms, this method generates activation maps that aid in the detection of re-entrant circuits and focal activities. In an early clinical ECGi mapping study, conducted in 2010, the presence of multiple wavelets was the most prevalent activation pattern in patients with AF with targeted, ECGi-guided ablation resulting in termination of AF [50]. Following that, the ECVUE ECGi mapping system (developed by CardioInsight, Cleveland, OH, USA) was employed to study 103 patients with persistent AF. The findings identified unstable re-entry circuits, rather than stable focal sources observed through FIRM mapping, as the primary mechanism for AF perpetuation in the patients studied [51, 52]. Subsequently the AFACART study (Multicentre evaluation of non-invasive biatrial mapping for persistent atrial fibrillation ablation), conducted in 2017 across multiple centers, investigated the effectiveness of ECGi-guided ablation in patients with persistent AF. The primary endpoint of the study was the termination of AF, with 77% of patients free from recurrence at the one-year mark [53]. It is important to note, however, that there are no randomized trials evaluating this technique in AF ablation.

The complexity involved in the transformation of electrograms obtained using phase mapping with limited ability for real-time intraprocedural analysis of raw signals are clear drawbacks to the phase-mapping approach. Additionally, limitations associated with electrode density and mapping resolution, as well as conflicting findings regarding the stability of drivers when comparing FIRM and ECGi mapping, have raised concerns regarding the reproducibility and validity of the technique. In recent years, additional mapping systems have emerged to enable the identification of AF drivers without relying on phase mapping transformation. Two notable systems are the Cartofinder contact mapping system (developed by Biosense Webster) and the AcQMap non-contact ultrasound-based mapping system (developed by Acutus Medical). Studies have shown that targeted elimination of focal or rotational activity identified using these platforms has been associated with high rates of AF termination and midterm success rates of up to 70% in patients with persistent AF [54, 55]. However, similar to ECGi mapping, there are currently no randomized trials available to assess the efficacy of these techniques. Further research is needed to evaluate and validate their utility in AF mapping and ablation procedures.

Collectively, when considering the findings of the studies mentioned above, and in alignment with the STAR AF II trial, there is no substantial evidence to support adjunct ablation strategies beyond PVI or ablation guided by mechanistic mapping techniques.

## 3. Where are We Now? —Recent Advances in the Field

The publication the landmark EAST-AFNET trial (Early Rhythm-Control Therapy in Patients with Atrial Fibrillation) in 2020 represented a paradigm shift in the management of AF [56]. In this multicentre trial of 2789 patients with early AF (median time from diagnosis of 36 days, >25% persistent) a marked reduction in adverse cardiovascular outcomes was observed in those randomized to early rhythm control with anti-arrhythmic drugs or catheter ablation vs usual care. Reduction in hard clinical endpoints and all-cause mortality with catheter ablation was additionally demonstrated in the CASTLE AF trial (Catheter Ablation for Atrial Fibrillation with Heart Failure) in predominantly persistent AF patients with heart failure [57]. The resulting emphasis on the value of pursuing sinus rhythm in selected patients, will inevitably translate to increased numbers referred for catheter ablation worldwide. While PVI remains the gold standard for paroxysmal AF, the debate continues regarding the optimal catheter ablation strategy for persistent AF.

### 3.1 Focus on Lesion Durability-Optimized Workflows for PVI

One possible explanation for the lower success rates observed in previous studies could be the failure to create durable transmural lesions during the initial procedure. This is reflected in the significant reduction in rates of pulmonary vein (PV) reconnection seen at redo procedures, after the advent of contact force sensing catheters [58, 59]. In recent years, increased emphasis has been placed on better understanding of the biophysics of ablation to maximise procedural efficacy through safe creation of transmural ablation scar. The Ablation Index (Carto3, Biosense Webster) is a metric which allows for real time assessment of lesion formation using the key parameters of contact force, time, and power combined in a weighted formula. Preclinical studies have demonstrated its ability to predict lesion depth [60] and in humans, the Ablation Index has shown promise in identifying sites of pulmonary vein reconnection during repeat procedure [61].

The “CLOSE protocol” workflow, which focuses on creating continuous and optimized RF lesions with specific target Ablation Index values, has shown promising outcomes in patients with paroxysmal AF with high rates of freedom from recurrence after a single procedure [62, 63]. In the PRAISE-AF study (Use of Ablation Index-Guided Ablation Results in High Rates of Durable Pulmonary Vein Isolation and Freedom From Arrhythmia in Persistent Atrial Fibrillation Patients), the CLOSE protocol was evaluated in a small group of patients with persistent AF. Following a protocol-mandated repeat procedure, with pulmonary vein re-isolation in 22% of patients demonstrating reconnections, 95% of patients were free from recurrent AF at one year [64].

These findings may suggest that optimized Ablation Index-guided PVI alone can achieve high clinical success rates in patients with persistent AF.

### 3.2 High Power Short Duration Ablation

Very high-power, short-duration ablation offers the potential advantage of enhanced procedural safety and efficacy, with pre-clinical studies suggesting predominantly resistive tissue heating, translating into a high rate of lesion contiguity and transmurality [65, 66, 67, 68]. In humans, the randomized POWER PLUS study (Very High-Power Ablation for Contiguous Pulmonary Vein Isolation) of PVI using 90 W/4 second vs conventional ablation with the QDOT Micro catheter (Biosense webster, Diamond Bar, CA, USA) in mixed population of paroxysmal and persistent AF patients confirmed a five-minute reduction in procedure time with a similar safety and midterm efficacy profile [69]. A trend towards lower first pass isolation was noted, with gaps frequently identified at the thick-walled anterior carina in the 90 W group. This is in line with a recent animal study reporting significantly smaller lesion sizes with this modality, reflecting a lower overall energy deposit to the tissue [70]. Further studies will shed light on the efficacy of very high-power ablation beyond the PVs, however the possibility of smaller lesions is a consideration when ablating at thicker regions such as the mitral isthmus.

### 3.3 Cryoablation for PVI in Persistent AF

An alternative form of energy delivery, cryoablation, has emerged over the last decade as a major competing technology with RF for PVI only. This ‘single-shot’ technique results in pulmonary vein isolation via delivery of a refrigerant to a custom-made balloon placed at the ostia of the pulmonary veins [71]. In paroxysmal AF patients it was found to be non-inferior in safety and efficacy to RF ablation in a large randomized controlled trial [72]. Several non-randomised studies have subsequently supported the feasibility of this approach in persistent AF patients, with modest results, comparable to those obtained with RF ablation [73, 74]. Results from the randomized, multicentre Fire and Ice II trial, comparing cryoballoon to RF ablation in persistent AF patients, are awaited [75].

### 3.4 Targeting of Atrial Fibrotic Remodelling 

While increased durability of PVI has translated into improved success rates in paroxysmal AF patients, in persistent AF patients with advanced substrate remodelling, it may not be a sufficient strategy to prevent recurrences.

Atrial fibrosis is a prominent feature of atrial structural remodeling, characterized by an excessive accumulation of extracellular matrix, as observed in histological studies. It increases with advancing age, can be present in cases of lone AF as well as AF associated with underlying cardiac conditions, and furthermore has been correlated with the severity and persistence of AF [76, 77, 78, 79]. Two methods exist for identifying and quantifying this important disease marker clinically: atrial late gadolinium enhancement cardiac magnetic resonance (LGE-CMR) imaging and invasive voltage mapping. The extent and severity of high signal intensity regions on LGE-CMR, or low voltage areas on invasive mapping (as surrogate markers for atrial fibrosis) has been shown to be a powerful independent predictor of recurrence post ablation [80, 81, 82]. Increased availability of LGE-CMR imaging and ongoing advances in catheter technology have allowed for the routine assessment of underlying fibrotic substrate in persistent AF patients before or during ablation, with the targeting of these areas for ablation a focus of recent trials.

Several studies have investigated the efficacy of voltage-guided ablation strategies, which involve ablating or isolating regions of low voltage in the atria. Rolf *et al*. [83] reported comparable success rates between patients who underwent voltage-guided ablation of low voltage areas and those without low voltage areas undergoing standard ablation. A subsequent small retrospective study demonstrated improved outcomes with additional voltage-guided ablation, specifically the posterior left atrial wall [12]. In the randomized, mulitcentre STABLE-SR study (Low-Voltage Myocardium-Guided Ablation Trial of Persistent Atrial Fibrillation) of persistent AF ablation examining the stepwise approach vs PVI plus homogenization of low voltage areas, comparable success rates were seen (74% vs 71%, *p* = 0.325) with lower procedure and fluoroscopy times in the group undergoing voltage guided ablation [84]. Several factors should be considered with voltage-guided ablation. Mapping resolution, relating directly to catheter electrode size and spacing, may influence the appearance of voltage maps, as can activation direction and local conduction velocity, which have been associated with changes in voltage amplitude in atrial and ventricular myocardium [85, 86]. Atrial rhythm may also affect recorded voltage which has been demonstrated to be lower during AF than sinus rhythm [87]. Furthermore, not all low voltage areas may be mechanistically relevant for atrial arrhythmogenesis. In the absence of robust histological validation for pre-ablation voltage mapping, optimum thresholds for defining relevant low voltage zones remain somewhat unclear. Most centres routinely apply a value of <0.5 mV to define low voltage, based on animal studies of post myocardial infarction scar in the ventricle [88, 89]. In a recent study Rillo *et al*. [90] identified an optimum range of 0.3–0.6 mV for identifying low voltage zones and surmised that only ‘non-compact’ zones are of interest for ablation. The comprehensive MASH II trial (Characterization of Atrial Substrate to Predict the Success of Pulmonary Vein Isolation: The Prospective, Multicenter MASH-AF II (Multipolar Atrial Substrate High Density Mapping in Atrial Fibrillation) Study) in 262 AF patients sought to further define low voltage zones and their association with outcome post ablation. It found that the impact of low voltage area on outcome differs between paroxysmal and persistent AF patients, suggesting that not all low voltage zones have the same prognostic implications [91].

**Fig. 1.**
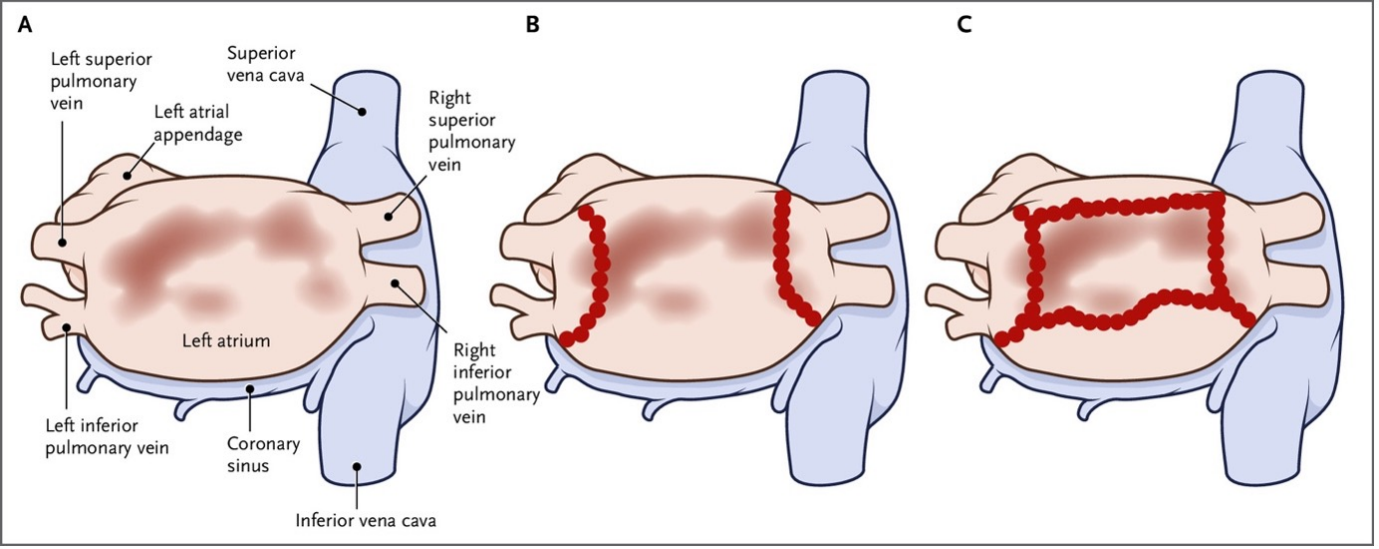
**Low voltage myocardium guided substrate ablation as employed in the ERASE trial**. (A) PA view of left atrium with low voltage areas identified in the posterior wall. (B) Ablation strategy in those randomized to PVI only. (C) Box isolation of left atrial posterior wall in addition to PVI as an example of strategy in those randomized to PVI plus substrate modification. Reproduced from Huo *et al*. ‘Low-Voltage Myocardium-Guided Ablation Trial of Persistent Atrial Fibrillation’. NEJM Evidence. 2022 Oct 25;1(11):EVIDoa2200141. Reprinted with permission from Massachusetts Medical Society. PVI, pulmonary vein isolation; PA, postero-anterior.

Regarding non-invasive CMR assessment of atrial substrate, the last two years has witnessed the publication of two important randomised trials examining adjunct fibrosis guided ablation using CMR or voltage mapping in persistent AF. The multicentre DECAAF II trial (Effect of MRI-Guided Fibrosis Ablation vs Conventional Catheter Ablation on Atrial Arrhythmia Recurrence in Patients With Persistent Atrial Fibrillation) randomised 843 persistent AF patients undergoing 1st time ablation to PVI vs PVI plus LGE-CMR guided atrial fibrosis ablation [8]. Ablation strategy in the latter arm included encircling or covering all areas of fibrosis identified on atrial LGE-CMR. After a follow up period of 12–18 months there was no difference in arrhythmia recurrence between groups (46 vs 43%, *p* = 0.63). Furthermore, more safety events occurred in patients undergoing additional fibrosis guided ablation (*p* = 0.001), particularly relating to a higher rate of ischaemic strokes in this group. Subsequently the multicentre ERASE trial (Low-Voltage Myocardium-Guided Ablation Trial of Persistent Atrial Fibrillation) randomised 324 persistent AF patients to PVI only vs PVI plus low voltage ablation based on the findings of invasive mapping [7] (Fig. 1). In contrast to the DECAAF study, the primary endpoint of arrhythmia recurrence of >30 seconds occurred in significantly more patients undergoing PVI only vs PVI plus voltage guided ablation (50 vs 35%, *p* = 0.006). It is worth noting that in this study, only those with low voltage areas underwent additional substrate ablation and that all those with ‘healthy’ atria underwent PVI only, regardless of randomisation group. As such only 54 out of the 324 patients randomised underwent voltage guided ablation. Furthermore, similar to DECAAF II a trend towards more complications were seen in the group randomised to additional substrate ablation. As for voltage mapping, lack of robust histological validation remains an inherent limitation. Several methods exist for thresholding atrial LGE images and the optimal threshold for identifying clinically relevant fibrotic regions to be targeted for ablation is still subject to debate. Inherent difficulties with imaging the very thin-walled atria and the requirement for bespoke software for image processing has limited the uptake of the technique to a relatively small number of expert centres, and differences in imaging protocols and thresholding techniques has resulted in difficulty reproducing results between centres.

Interestingly, using both invasive and non-invasive measures of fibrosis characterisation, high success rates with PVI only, of up to 84%, were seen in sub-analyses of the STABLE SR and DECAAF II trials in patients without evidence of atrial fibrosis [8, 84]. This again underscores the point that in persistent AF patients with early-stage disease, PVI alone is likely to be a sufficient strategy.

### 3.5 Novel Techniques for Persistent AF Ablation

#### 3.5.1 Epicardial Ablation

In addition to fibrosis-guided ablation, there has been increasing interest in targeting epicardial structures as an adjunctive strategy in the treatment of persistent AF. One such structure that has gained attention is the Vein of Marshall (VOM), a branch of the great cardiac vein and an embryological remnant of the superior vena cava. The VOM has been demonstrated as a source of AF triggers and is known to harbor sympathetic and parasympathetic and nerve fibers that play a part in the pathogenesis and maintenance of AF [92, 93]. Its anatomic location at the mitral isthmus also makes it an important structure for facilitating mitral isthmus block. The VENUS-AF trial (Effect of Catheter Ablation With Vein of Marshall Ethanol Infusion vs Catheter Ablation Alone on Persistent Atrial Fibrillation), published in 2020, aimed to investigate the efficacy of adjunctive VOM ethanol infusion during catheter ablation for persistent AF. The trial randomized 343 patients into two groups: catheter ablation alone and catheter ablation plus VOM ethanol infusion [82]. At 6- and 12-month follow-up, those receiving adjunct VOM ethanol infusion had significantly higher freedom from recurrent atrial tachyarrhythmia compared to the ablation-alone group (*p* = 0.04). On secondary analysis, the presence of mitral isthmus block was a significant predictor of a successful outcome post-ablation [94]. However, it is important to note that overall atrial tachy-arrhythmia-free survival rates were modest (65.2% vs 53.8%) and substantial additional substrate ablation was performed in both groups, limiting a robust evaluation of the added value of VOM ethanol infusion. Subsequently, the randomized Marshall plan study investigated a comprehensive strategy of PVI alone vs PVI plus linear ablation at the cavo-tricuspid isthmus (CTI), roof and mitral isthmus and VOM ethanol infusion. Preliminary 10-month follow-up results, presented recently, demonstrated significantly higher success rates in those receiving the Marshall plan strategy vs PVI alone (87 vs 70%) [95]. This was a single centre trial, however, in a relatively small number of patients (n = 120). Confirmation of benefit at final follow-up with replication of similar success rates in larger multicentre trials is needed before drawing strong conclusions from this data and it should be noted that procedure times tend to be lengthy with the above strategy. Regardless of its impact on post-ablation outcomes, it is undoubtedly the case that VOM ethanol infusion greatly facilitates mitral isthmus block. This is evidenced by a significantly greater rate of acute mitral isthmus block (98.7% vs 63.6%) in a comparative study of 262 patients undergoing adjunct VOM vs RF only ablation, with higher rates of persistent block seen at repeat procedure [96]. Furthermore, performing VOM ethanol infusion as a first step reduced the amount of RF applications needed to achieve acute block in a small randomized study [97]. In summary, although the use of adjunct VOM ethanolization during catheter ablation has shown improved rates of mitral isthmus block, more research is needed to fully understand its potential additional benefit on outcomes in patients with persistent AF.

More extensive epicardial ablation strategies, facilitated by minimally invasive surgical access have also been evaluated in this population, particularly in those with long-standing persistent AF. The rationale for this approach is facilitation of extensive, durable, and transmural lesions with the benefit of enhanced oesophageal safety when performing posterior wall lesions. Furthermore endocardial-epicardial dissociation is well described in advanced AF, creating a 3-dimensional (3D) substrate for arrhythmia [98] that may not be adequately targeted by endocardial ablation alone. The CONVERGE trial (Hybrid Convergent Procedure for the Treatment of Persistent and Long-Standing Persistent Atrial Fibrillation) randomized 153 persistent AF patients to a hybrid procedure involving minimally invasive epicardial ablation of the pulmonary veins and posterior wall with subsequent endocardial ablation (consisting of a CTI line with ‘touch up’ ablation of the left atrial lesion set if needed) vs a standard endocardial ablation consisting of PVI, roof and CTI lines [99]. Freedom from ATA at 12 months was achieved in substantially more patients in the hybrid vs the conventional endocardial arm (67.7 vs 50%, *p* = 0.036) with more safety events in the hybrid group. Furthermore, differences in lesion sets between groups, with posterior wall isolation not performed in the endocardial group, should be a consideration when interpreting success rates in these two populations. Very recently, preliminary results from the CEASE-AF (Efficacy and safety of hybrid epicardial and endocardial ablation versus endocardial ablation in patients with persistent and longstanding persistent atrial fibrillation: a randomised, controlled trial) multicentre, randomized controlled trial, comparing a staged hybrid ablation approach to conventional endocardial ablation in persistent and long-standing persistent AF up to 10 years reported a 32.4% absolute benefit increase in effectiveness, with 71.6% of patients in the hybrid group free from atrial tachyarrhythmia (ATA) at one year [100]. Although there was no difference between groups, complication rates were high in both arms at 7.8% vs 5.8%. Criticisms include low success rates in the endocardial ablation arm with, again, a significantly more aggressive lesion set in the hybrid approach. Additionally, early results from the recently completed HART-CAP (Hybrid Versus Catheter Ablation in Persistent Atrial Fibrillation) randomized trial reported an 89% freedom from AF in patients undergoing a hybrid approach vs 41% undergoing endocardial ablation (*p* = 0.002) with a 5% major complication rate in both arms [101]. This was a small study and as for CEASE AF, the rate of major adverse events in the endocardial group was higher than would be expected. Long procedure times and higher complication rates should be considered and longer follow up studies are needed to assess for a sustained benefit with this approach over time.

#### 3.5.2 Pulsed Field Ablation 

**Fig. 2.**
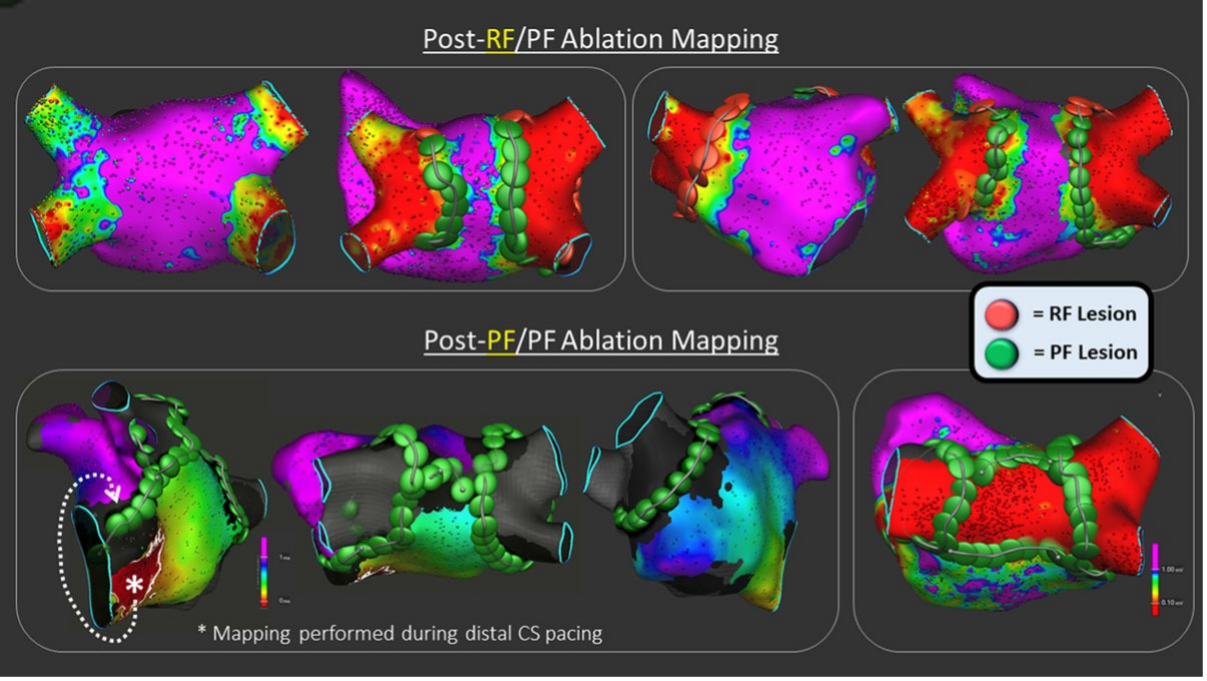
**Examples of PVI only (top panels) and PVI plus additional substrate modification (linear lesions and box isolation, bottom panels) using a lattice tip catheter capable of both RF and PFA delivery**. Reproduced from ‘Lattice-Tip Focal Catheter That Toggles Between Radiofrequency and Pulsed Field Energy to Treat Atrial Fibrillation, A First-in-Human Trial’. Circ Arrhythm Electrophysiol. Reprinted with permission from Wolter Kluwer Health* (*The Creative Commons license does not apply to this content. Use of the material in any format is prohibited without written permission from the publisher, Wolters Kluwer Health, Inc. Please contact permissions@lww.com for further information). PVI, pulmonary vein isolation; RF, radiofrequency; PFA, Pulsed Field Ablation; PF, pulsed field; CS, coronary sinus.

The advent of Pulsed Field Ablation (PFA) represents one of the most exciting developments in the field of catheter ablation in the last decade. Unlike RF ablation, PFA represents a non-thermal based energy modality whereby delivery of a rapid sequence of high amplitude electrical pulses causes cell death through electroporation of the sarcolemmal membrane. It preferentially targets myocardial cells, offering a safety advantage due to sparing of adjacent tissue, at risk of damage during conventional RF ablation from collateral heating. Indeed, the safety of this modality has been demonstrated consistently on pre-clinical studies with no evidence of collateral tissue damage [102, 103, 104]. The preliminary IMPULSE (A Safety and Feasibility Study of the IOWA Approach Endocardial Ablation System to Treat Atrial Fibrillation) and PEFCAT studies (A Safety and Feasibility Study of the FARAPULSE Endocardial Ablation System to Treat Paroxysmal Atrial Fibrillation), investigating the commercially available Farapulse PFA system and multipsline catheter (Farapulse, Menlo Park, CA, USA) for “single shot” PVI in individuals with paroxysmal AF, reported an impressive 100% durability of PVI after three months, accompanied by an excellent safety profile [105]. A subsequent study in persistent AF patients employed the same catheter for additional substrate ablation at the posterior wall [106]. In all cases the pulmonary veins and posterior wall were acutely isolated with persistent posterior wall isolation in 100% at repeat, protocol-mandated procedure at 75 days. Following on from this the Pulsed-AF prospective multicentre trial reported on safety and efficacy outcomes in 150 paroxysmal and 150 persistent AF patients undergoing PVI only using the Medtronic PFA system and circular ‘single shot’ PFA catheter (PulseSelect, Medtronic, Minneapolis, MN, USA) [107]. At one year success rates using weekly, trans-telephonic monitoring were 55% in the persistent arm and 66% in paroxysmal AF patients. Clinical success rates based on freedom from symptomatic arrhythmia were higher at approximately 80% in both groups and in line with the pre-clinical and early clinical studies reported above. Again, complication rates were very low with no oesphageal, PV or phrenic nerve injury. Distinct from the single shot catheters detailed above, a lattice tip catheter capable of ‘large footprint’, focal PFA and RF delivery (Affera, Medtronic, Minneapolis, MN, USA), has demonstrated promise in recent studies with very good efficacy and safety in patients undergoing PVI and adjunct roof, mitral and CTI ablation [108, 109] (Fig. 2). A recently published study from the same group in a larger population including 108 persistent AF patients reported on outcomes when this catheter was used for extensive substrate ablation in addition to PVI including 78 mitral, 121 CTI and 130 roof lines [110]. Using a combination of PF and RF ablation, pulmonary vein isolation and linear block was achieved in 100% of patients with high rates of durability seen on invasive remapping. At one year follow-up, freedom from arrhythmia was similar for both paroxysmal and persistent AF patients (78.3 ± 6.0% and 77.9 ± 4.1%, respectively). Ongoing randomized studies will reveal whether initial promise will translate into a robust clinical benefit to the patient on long-term follow up. Nevertheless, the advantages in terms of safety and versatility coupled with lower procedural times make this catheter and energy delivery an attractive alternative for performing substrate ablation in a safe and efficient manner in the persistent AF cohort.

## 4. Re-Defining Outcome Measures and Improving Trial Design

### 4.1 Scope for Improved Classification of AF 

The traditional 7-day AF classification does not capture the true extent of underlying arrhythmia substrate in the persistent AF cohort. Heterogeneity in disease subtype may be better characterised by more specific markers of atrial remodelling including atrial size and presence of fibrosis, duration of continuous AF episodes, rhythm at time of procedure and number of and response to prior cardioversions. Significant variation exists, however, regarding the consistent reporting of such parameters in studies and therefore subtype of AF is likely to differ between trials rendering direct comparison of results difficult. The refinement of AF definition coupled with the reporting of a wide range of such disease related parameters will be important for future trial design to enhance interpretation of results and to identify or correct for selection bias.

### 4.2 Re-Defining Measures of Success

When evaluating trial outcomes, it is crucial to take into account the techniques utilized for post-procedural rhythm monitoring. Greater sensitivity in detection of arrhythmia recurrence is seen with increasing intensity of rhythm monitoring [111] however there is significant variation among studies in terms of the duration and approach to follow-up monitoring. Consistent between most studies to date, is the use of the endpoint of >30 seconds of atrial tachyarrhythmia to define arrhythmia recurrence, but this parameter may underestimate the clinically relevant benefit derived by the patient from ablation. As such, AF burden, defined as the percentage of time in AF, and most accurately measured with implantable cardiac monitors (ICM), has been increasingly employed as an outcome measure. This parameter is associated with symptomatology and quality of life [112, 113] as well as hard clinical outcomes including heart failure, stroke and mortality [57, 114, 115, 116] and may represent a more meaningful marker of success post ablation [117]. In the CAMERA-MRI study (Catheter Ablation Versus Medical Rate Control in Atrial Fibrillation and Systolic Dysfunction), ICM (implanted at time of procedure)-determined atrial tachyarrhythmia burden at 6 months post ablation was 1.5% in persistent AF patients with high rate of prior cardioversion, despite an overall freedom from recurrence of only 56% [118]. Similarly in the CASA-AF trial (Catheter ablation vs. thoracoscopic surgical ablation in long-standing persistent atrial fibrillation), 77% of long-standing persistent AF patients undergoing ICM implantation at the time of ablation, demonstrated a reduction in AF burden >75% despite an only 28% freedom from recurrence [119]. The CLOSEMAZE trial (Impact of Catheter Ablation on Arrhythmia Burden in Patients With Shock-Resistant Persistent Atrial Fibrillation) assessed the impact of catheter ablation on AF burden through ICMs implanted 2 months pre-procedure and noted a median atrial tachyarrhythmia burden reduction of 100%, despite an overall modest arrhythmia free survival of 55%, adding weight to the argument that conventional arrhythmia-free survival analysis does not capture the true impact of catheter ablation in this patient cohort [120] (article in press, JACC EP 2023, Fig. 3).

**Fig. 3.**
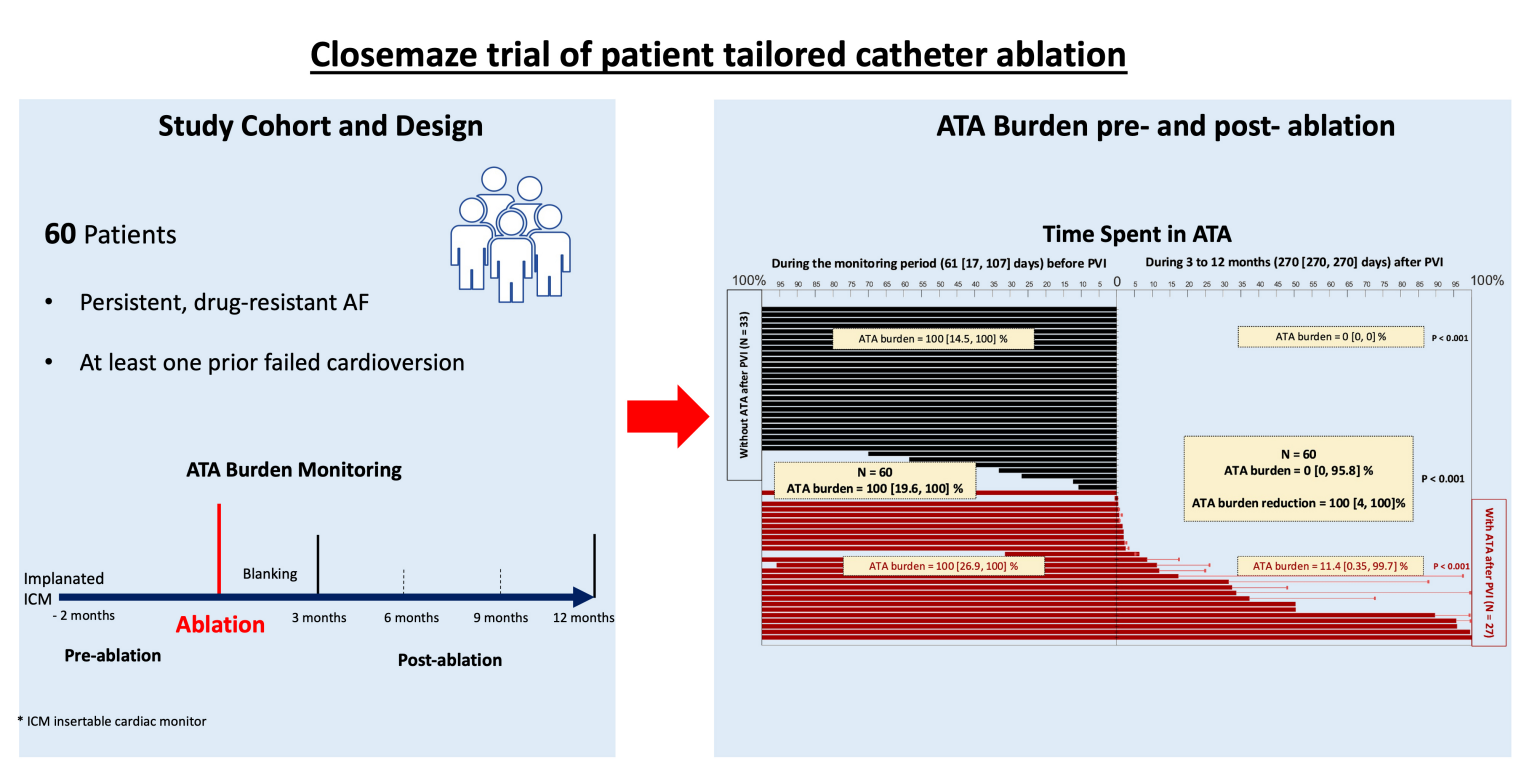
**Results from the Closemaze trial in persistent AF patients undergoing patient-tailored catheter ablation**. (Left panel) patient population and monitoring regime. (Right panel) Plot demonstrating ATA burden on implantable cardiac monitoring before and after ablation, with a significant reduction in burden post-ablation. ICM, implantable cardiac monitor; AF, atrial fibrillation; ATA, atrial tachyarrhythmia; PVI, pulmonary vein isolation.

### 4.3 Patient Selection and Tailored Ablation 

The diverse range of atrial remodeling observed in patients with persistent AF indicates that a personalized or “patient-tailored” approach to ablation may be more suitable than a generic “one size fits all” approach for this patient population. This approach is employed to some degree in the ERASE and DECAAF II trials whereby ablation strategy is dependent on the location and extent of low voltage or fibrotic regions on MRI. The heterogeneity of ablation strategies employed in these trials can be a limitation when interpreting results, however. In our centre, the choice of ablation strategy at index procedure in persistent AF patients is influenced by AF burden and the presence of parameters suggestive of non-PV triggered AF. Patients without significant atrial remodelling and with self-terminating AF are classified as having ‘pseudo’ persistent AF and undergo PVI only without adjunct ablation [3]. Those who do not fulfill the above criteria are classified as having truly persistent AF and undergo additional substrate ablation as an adjunct to PVI with linear ablation at the roof and mitral isthmus as well as Vein of Marshall ethanolisation. Ongoing randomized trials of patient tailored ablation and improved patient selection for adjunct strategies will be needed to optimise results in those with more advanced disease.

## 5. Conclusions

New evidence for clinical benefit with early rhythm control strategies will result in increased numbers of persistent AF patients referred for catheter ablation. While PVI may be sufficient in early persistent AF patients without significant atrial remodelling, those with more advanced, substrate-driven AF may require additional ablation beyond the pulmonary veins. Nevertheless, despite the extensive amount of prior research on additional substrate-based ablation techniques for persistent AF, none has consistently demonstrated an advantage when compared to PVI alone, and the pivotal role of PVI in all AF subtypes is emphasised in recent guidelines. Recent advancements in catheter technology, combined with refined ablation workflows, have led to improved durability of PVI and concurrently, the introduction of PFA represents a promising development in recent years that may improve efficacy and safety outcomes in the persistent AF cohort. Coupled with these technological developments, patient outcomes may be further improved through ongoing advancements in substate assessment and understanding of mechanistic processes to allow for optimisation of ablation strategy on an individualized basis.
